# Diabetic limb salvage procedure with bone allograft and free flap transfer: a case report

**DOI:** 10.1080/2000625X.2016.1270076

**Published:** 2017-01-16

**Authors:** Alexandre L. Godoy-Santos, Daniel T. Amodio, André Pires, Ana L. M. Lima, Teng H. Wei, Cesar de Cesar-Netto, David G. Armstrong

**Affiliations:** ^a^ Department of Orthopedic Surgery, University of São Paulo, São Paulo, Brazil; ^b^ Department of Orthopedic Surgery, Hospital do Coraçao, São Paulo, Brazil; ^c^ Department of Orthopedic Surgery, Medstar Union Memorial Hospital, Baltimore, MD, USA; ^d^ Department of Surgery, The University of Arizona, Tucson, AZ, USA

**Keywords:** Diabetic foot, reconstruction, free flap, soft-tissue allograft

## Abstract

The aim of this case report was to describe a successful diabetic limb salvage procedure in the treatment of an infected diabetic foot ulcer through a multidisciplinary team approach and complex surgical reconstruction involving a femoral head bone allograft and musculocutaneous latissimus dorsi free flap. The decision to proceed with aggressive staged efforts at diabetic limb salvage should be made only after careful consultation with the patient, his or her family, and the rest of the multidisciplinary healthcare team.

## Introduction

Diabetes mellitus has a global prevalence of 8.3%, and approximately 387 million people have been diagnosed worldwide. These numbers are expected to increase by 55% over the next 20 years. Global costs related to its treatment in the USA account for 612 billion USD annually, representing 11% of the total healthcare spending on adults [1]. Each year, 23 million people with diabetes mellitus develop a diabetic foot ulcer (DFU). This common complication often leads to significant functional loss, decreased quality of life, and socioeconomic impairment, affecting patients, their families, and the healthcare system [1]. The annual costs for the treatment of diabetic limb complications in the USA are around 17 billion USD, which is more than the direct costs of the five most costly cancers in the USA [2,3].

DFU can also lead to limb amputation, with annual costs of 200 million USD [4,5] in the USA and £55 million in England [6]. The chance of a particular patient having the contralateral limb amputated is 50% within a period of 2 years [7–9].

Diabetic limb salvage procedures may represent a feasible and more functional option in the treatment of these patients, who often have associated diseases that already negatively impact their quality of life and life expectancy [10,11].

A multidisciplinary approach and medical advances in the past two decades have improved the ability to reconstruct complicated and infected diabetic lower extremities. Feet that once would have been amputated can now be managed by salvage procedures, with similar or even better functional outcomes compared to diabetic patients who undergo amputation [12,13]. In this scenario, it is fundamental to consider the option of a surgical reconstruction, rather than an amputation, when treating diabetic patients with complicated and infected DFUs [14].

In this article, we present a case report of a successful limb salvage procedure in the treatment of an infected DFU. The pattern of injury was indicative of neuropathic etiology, confirmed by a monofilament test and electromyography. A multidisciplinary approach was taken, with complex surgical reconstruction involving a femoral head bone allograft and musculocutaneous latissimus dorsi free flap.

## Case report

**Figure 1.**
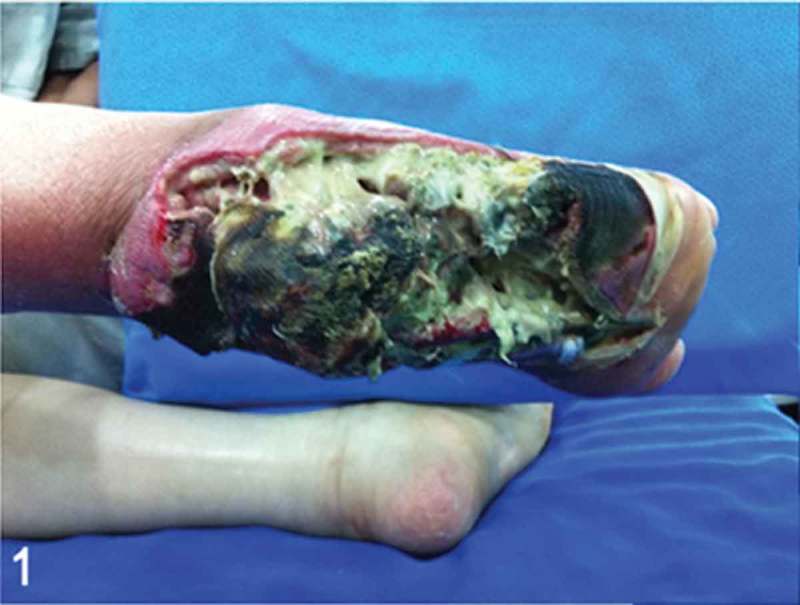
Clinical picture of the infected foot during admission to the emergency room. Note the extensive plantar fat pad and soft-tissue necrosis, calcaneal bone exposure, purulent drainage, forefoot necrosis, and epidermolysis.

A 54-year-old female with uncontrolled type 2 diabetes mellitus presented to our emergency room facility with respiratory insufficiency and hemodynamic instability secondary to a progressive infected diabetic heel ulcer. Physical examination revealed a deep, necrotic, and infected plantar heel ulcer with exposure of the calcaneus bone, flexor hallucis longus, and flexor digitorum longus tendons, without any signs of peripheral arterial disease. The patient had been seen in the clinic 6 weeks earlier and at that point had only a small, non-infected heel wound that was treated with local debridement and dressing changes. Figure 1 shows the clinical presentation of the infected foot when the patient was seen in our emergency facility.

### Detailed clinical treatment

**Day 1**: In the intensive care unit, medical support measures for patients in critical condition were performed, with intravenous fluid expansion, metabolic and blood glucose control, vasopressor medications, and invasive mechanical ventilation. Broad-spectrum antibiotic therapy was started with the intention of narrowing it after culture results [15]. Tight blood glucose control was initiated, with a combination of regular, intermediate-acting, and long-acting insulin. Desired blood glucose levels were 70–130 mg/dl before meals and 180 mg/dl after meals, with a hemoglobin A1c of 6%.

**Day 3**: After significant clinical improvement, mechanical ventilation was interrupted and the patient was transferred to the inpatient ward unit.

**Day 7**: Soft-tissue cultures and antibiotic susceptibility results are presented in Tables 1 and 2. The patient’s antibiotic therapy was narrowed following these results. She was treated with a combination of intravenous vancomycin, meropenem, and fluconazole.

**Table 1.** Bacterial distribution cultures and susceptibility tests.

*Staphylococcus coagulase-negative*		*Acinetobacter baumannii* complex		*Enterobacter cloacae* complex	
Amoxicillin–clavulanic acid	R	Amikacin	R	Amikacin	R
Cephalothin	R	Cefepime	R	Ampicillin–sulbactam	R
Cefazolin	R	Ceftazidime	R	Cefepime	R
Ciprofloxacin	R	Ceftriaxone	R	Ceftazidime	R
Clindamycin	R	Ciprofloxacin	R	Ceftriaxone	R
Gentamicin	R	Gentamicin	I	Ciprofloxacin	R
Erythromycin	R	Imipenem	R	Gentamicin	I
Gentamicin	R	Meropenem	S	Imipenem	R
Linezolid	S	Piperacillin–tazobactam	R	Meropenem	S
Oxacillin	R	Sulfamethoxazole–trimethoprim	R	Piperacillin–tazobactam	R
Sulfamethoxazole–trimethoprim	R	Polimyxin B	S	Sulfamethoxazole–trimethoprim	R
Tetracycline	S				
Teicoplanin	S				
Vancomycin	S				

R = resistant; I = intermediate; S = susceptible.

**Table 2.** Fungal distribution cultures and susceptibility tests.

*Candida glabrata*		*Candida albicans*	
Amphotericin b	0.50 (S)	Amphotericin b	1.0 (S)
Caspofungin	0.50 (S)	Caspofungin	0.125 (S)
Fluconazole	≥ 64 (S)	Fluconazole	≤ 1.0 (S)
Voriconazole	1.0 (S)	Voriconazole	≤ 0.12 (S)

S = susceptible.

**Week 8**: Her intravenous antibiotic therapy was discontinued.

### Detailed surgical treatment

A staged surgical treatment protocol was performed based on the following procedures.

**Figure 2.**
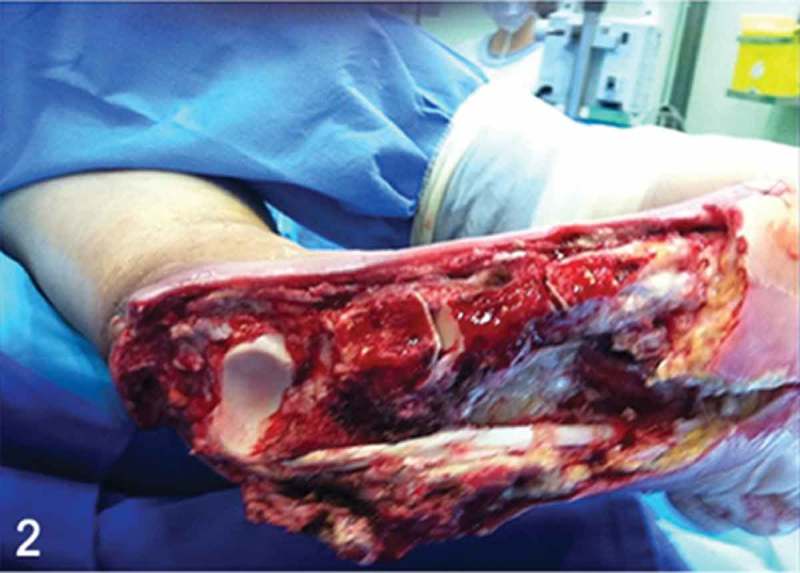
Clinical picture after the first surgical irrigation and debridement. Note the aggressive resection of all muscular structures of the central and lateral compartments of the foot, peroneal tendons, partial bone resection of the cuboid and fifth metatarsal base, and almost complete resection of the calcaneal bone (the anterior process of the calcaneus was maintained). Note the healthy exposed talar undersurface of the posterior facet of the subtalar joint.

**Figure 3.**
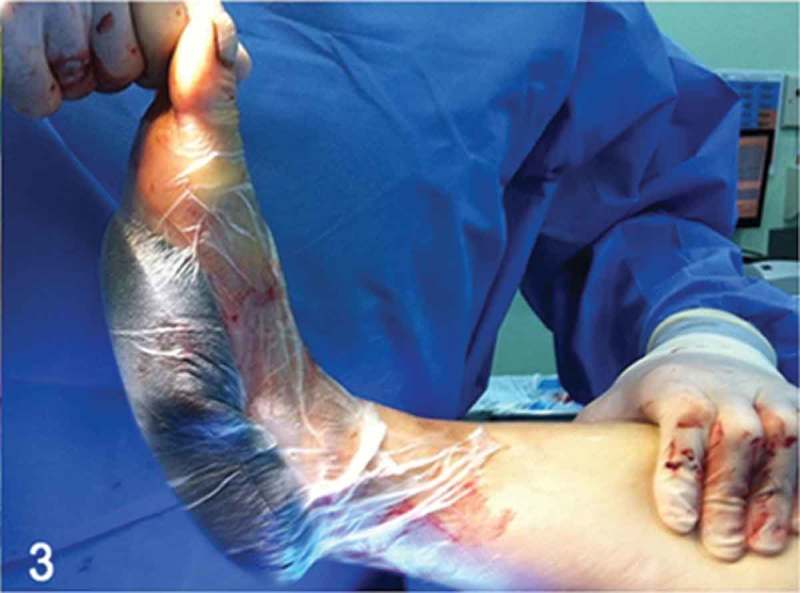
Negative pressure wound therapy application to the wound following the first surgical debridement.

**Day 1**: Irrigation and debridement of all devitalized and infected tissue was performed. Specimens were collected for culture and antibiotic susceptibility testing. Provisional negative pressure wound therapy (ActiV.A.C.®; KCI, San Antonio, TX, USA) was installed (125 mmHg) and set to be continuous for the first 24 h and intermittent in the next 48 h (Figures 2 and 3).

**Figure 4.**
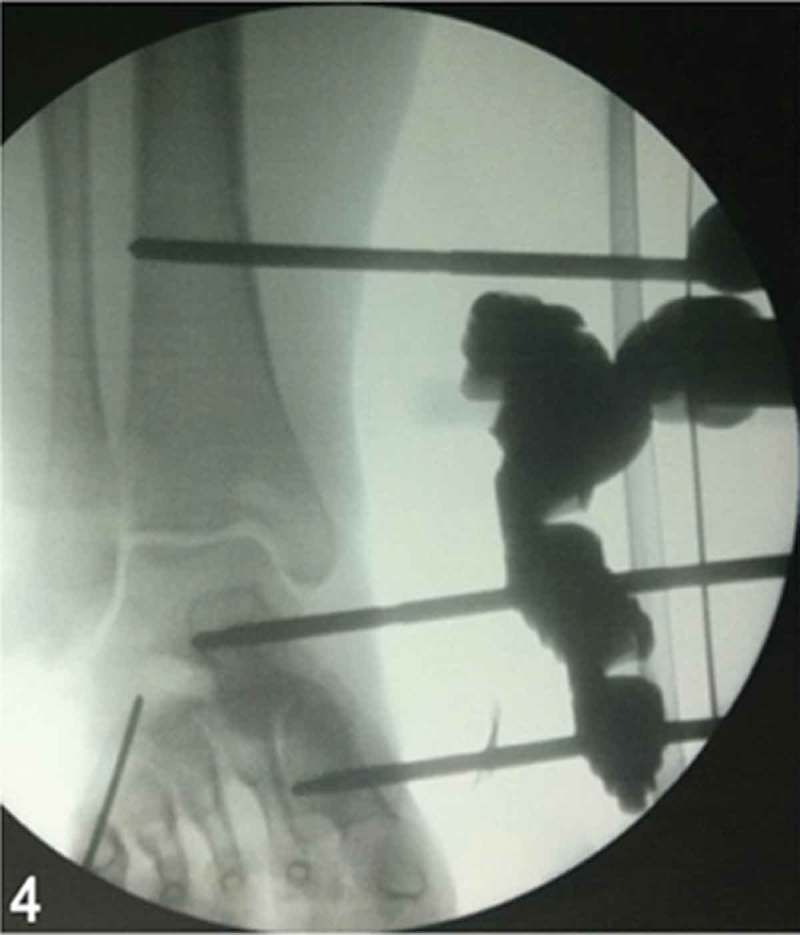
Intraoperative anteroposterior ankle fluoroscopic image. External fixation was performed to maintain a neutral positioning of the ankle and to allow stable soft-tissue healing. Four Schanz half-pins, two in the tibia proximally, one in the neck of the talus, and one in the base of the first metatarsal, were applied and connected.

**Day 3**: A second irrigation and surgical debridement was performed, with additional tissue sent for culture and antibiotic susceptibility testing. No macroscopic evidence of progressive infection or necrosis was observed. At that time, an external fixation was applied to maintain the ankle at 90° in the sagittal plane and a percutaneous fixation with Kirschner wire was used to maintain the correct relationship between lateral and medial columns of the foot (Figure 4). A new negative pressure wound therapy was applied at 125 mmHg, set to be continuous for the first 24 h and intermittent in the following 3 days.

**Figure 5.**
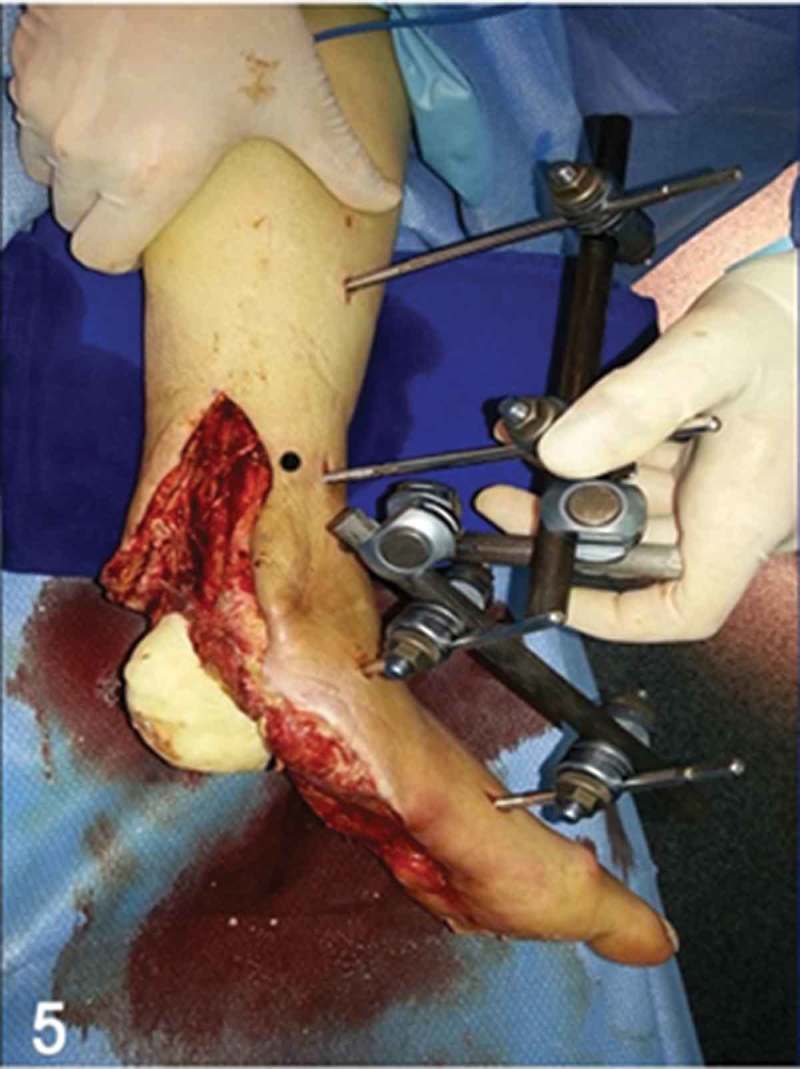
Clinical picture of the surgical procedure of hindfoot reconstruction. Note the antibiotic-loaded polymethylmethacrylate bone cement positioned where once was the calcaneal bone, underneath the talar posterior facet of the subtalar joint.

**Figure 6.**
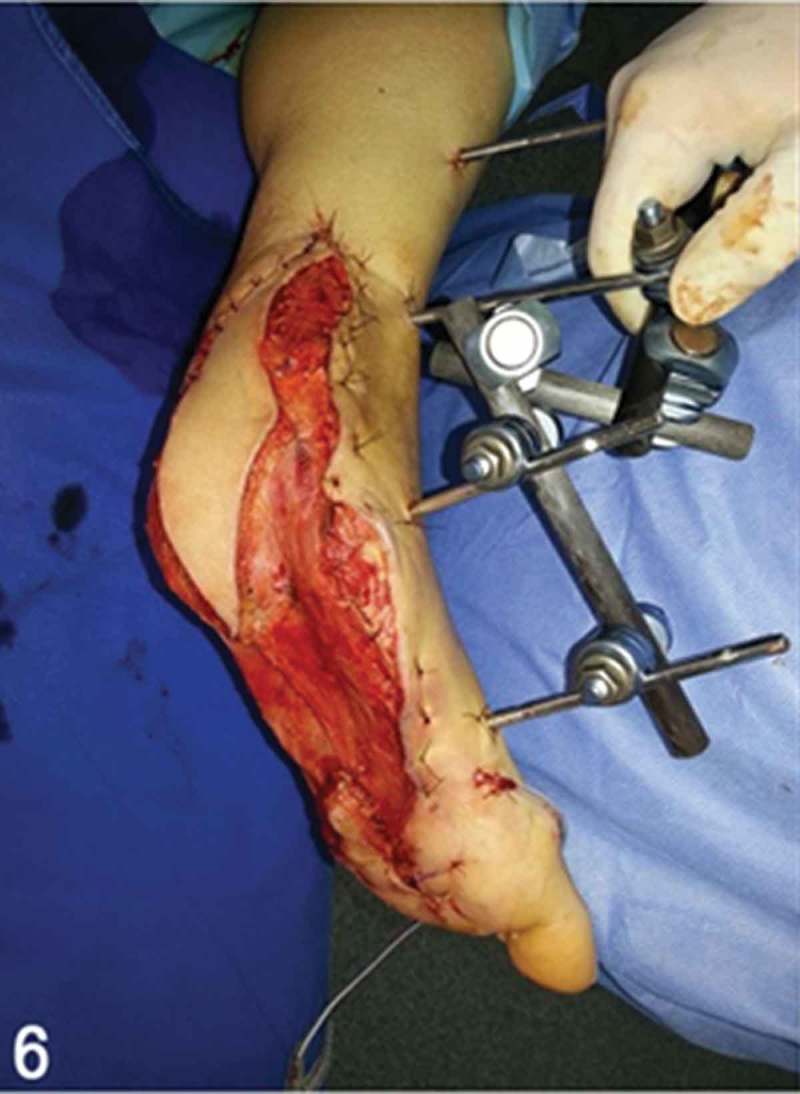
In the same surgical procedure, the heel soft-tissue coverage was achieved through a free musculocutaneous latissimus dorsi flap.

**Day 7**: Forty grams of vancomycin-loaded polymethylmethacrylate bone cement (Surgical Simplex P®, Miami, FL, USA) was used to fill the void of the calcaneus bone removed in prior surgical debridements (Figure 5). After that, a heel soft-tissue coverage procedure was performed. We used a free musculocutaneous latissimus dorsi flap (Figure 6) for soft-tissue coverage of the heel defect. The choice of this specific soft-tissue coverage was made secondary to characteristics of the injury, which included the large defect and location (plantar surface) that needed to be addressed, and also based on the unique features of this flap. This flap possesses excellent skin properties of the donor site (posterior surface of the trunk) with a thinner composition than other fasciocutaneous flaps, allowing plantar support and weight-bearing in the hindfoot and facilitating gait and proper fit in shoe-gear. End-to-end microanastomosis was performed between the thoracodorsal and posterior tibial arteries.

**Figure 7.**
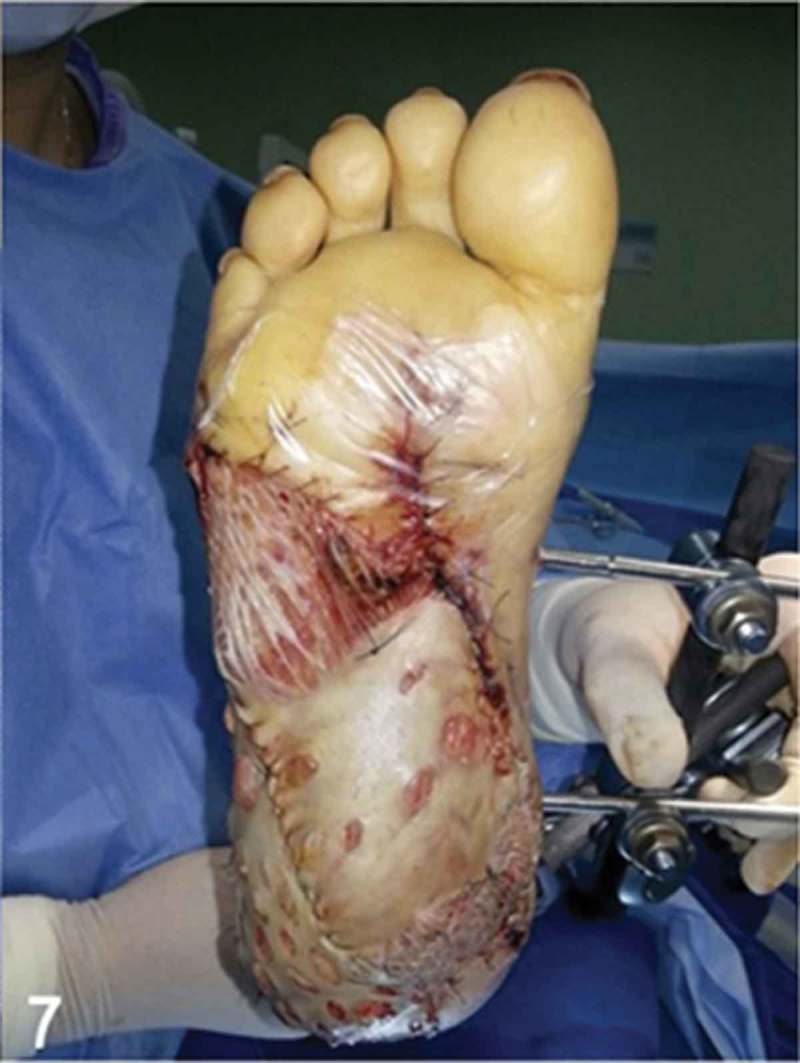
After adequate monitoring of the viability of the free musculocutaneous flap, multiple split-thickness skin grafts from the thigh were performed to cover the free muscle and granulation tissue surfaces on the heel and plantar aspect of the foot.

**Day 12**: After adequate monitoring of the viability of the free musculocutaneous flap, and making certain that no venous insufficiency, venous stasis, or necrosis occurred in the flap, a split-thickness skin graft from the thigh to cover the free muscle and granulation tissue surfaces was performed (Figure 7).

**Figure 8.**
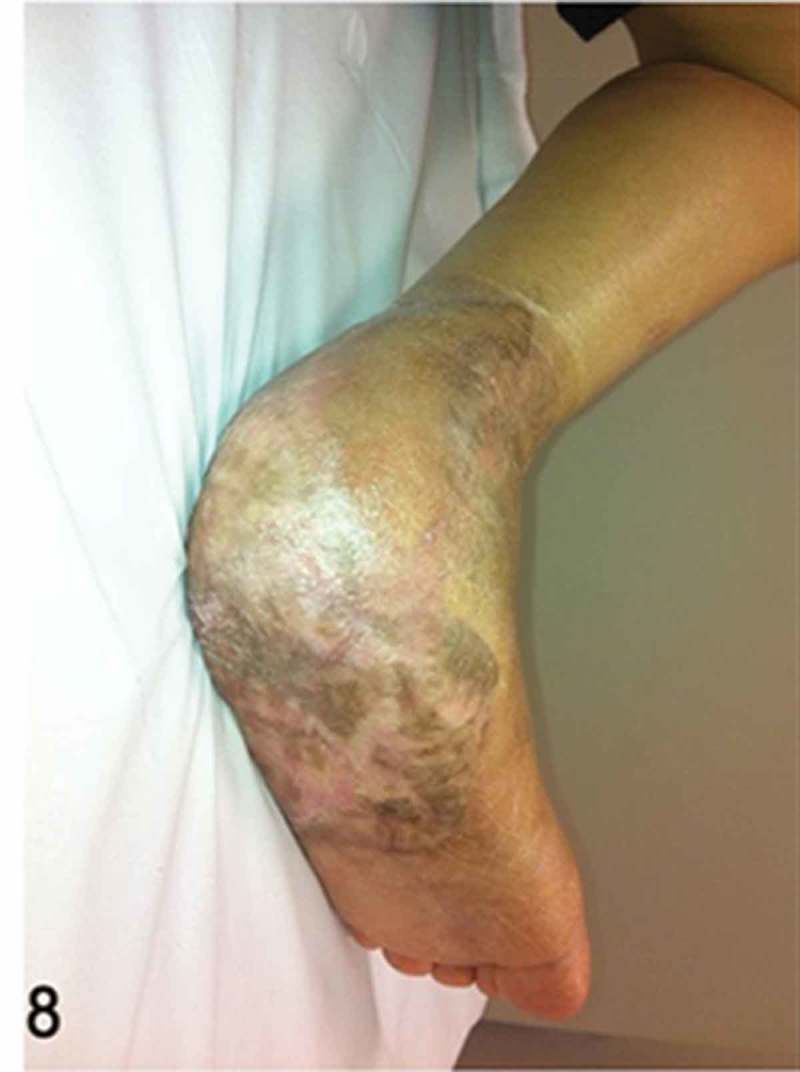
Clinical picture of the patient’s foot in a follow-up appointment at 12 weeks. The free flap and skin graft were fully integrated.

**Figure 9.**
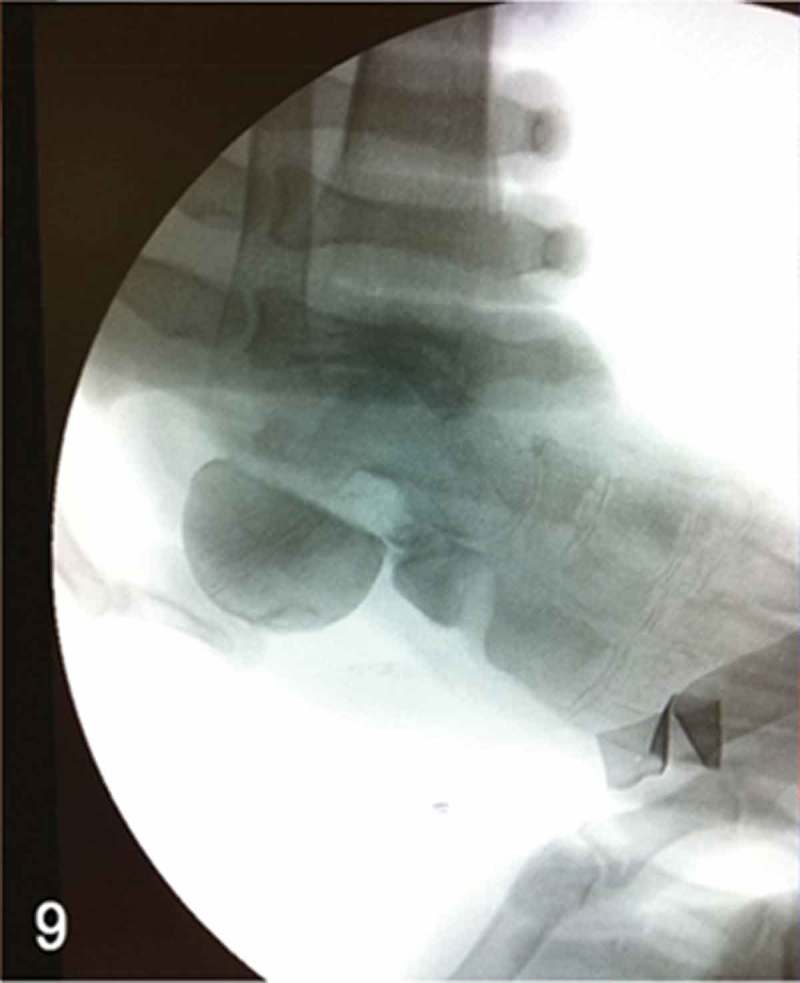
Intraoperative lateral foot fluoroscopic image, demonstrating the positioning of the femoral head bone allograft. Articular cartilage of the subtalar joint was prepared, aiming for an arthrodesis between the bone allograft and talar bone.

**Figure 10.**
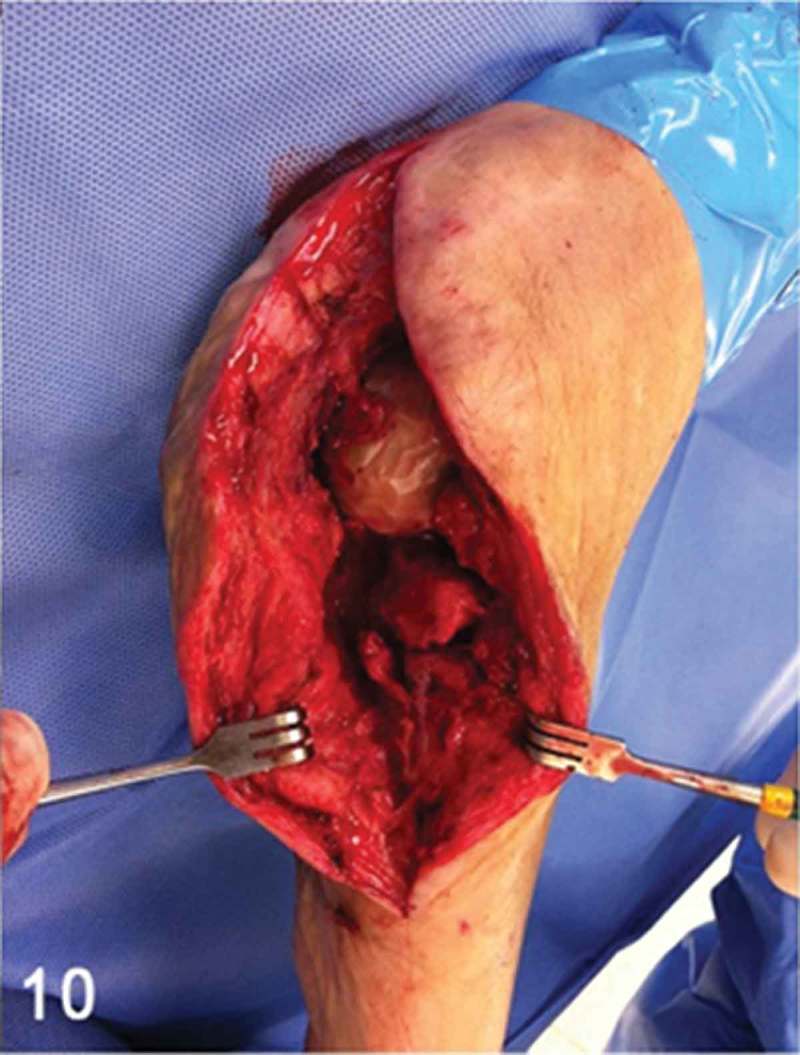
Full-thickness posterolateral approach was performed, parallel to the edge of the free flap. Note the femoral head bone allograft and the anterior process of the calcaneus bone.

**Figure 11.**
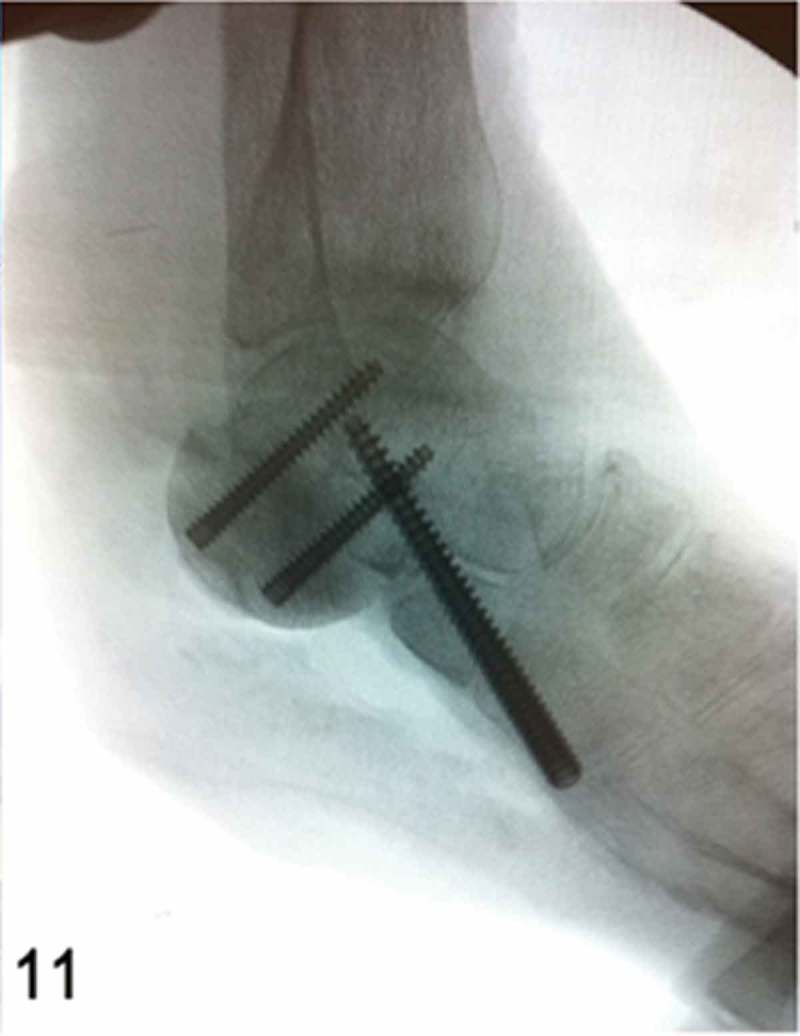
Intraoperative lateral foot fluoroscopic image, showing the internal fixation arthrodesis procedure using headless compression screws between the femoral head bone allograft, talus, anterior process of the calcaneus and cuboid.

**Figure 12.**
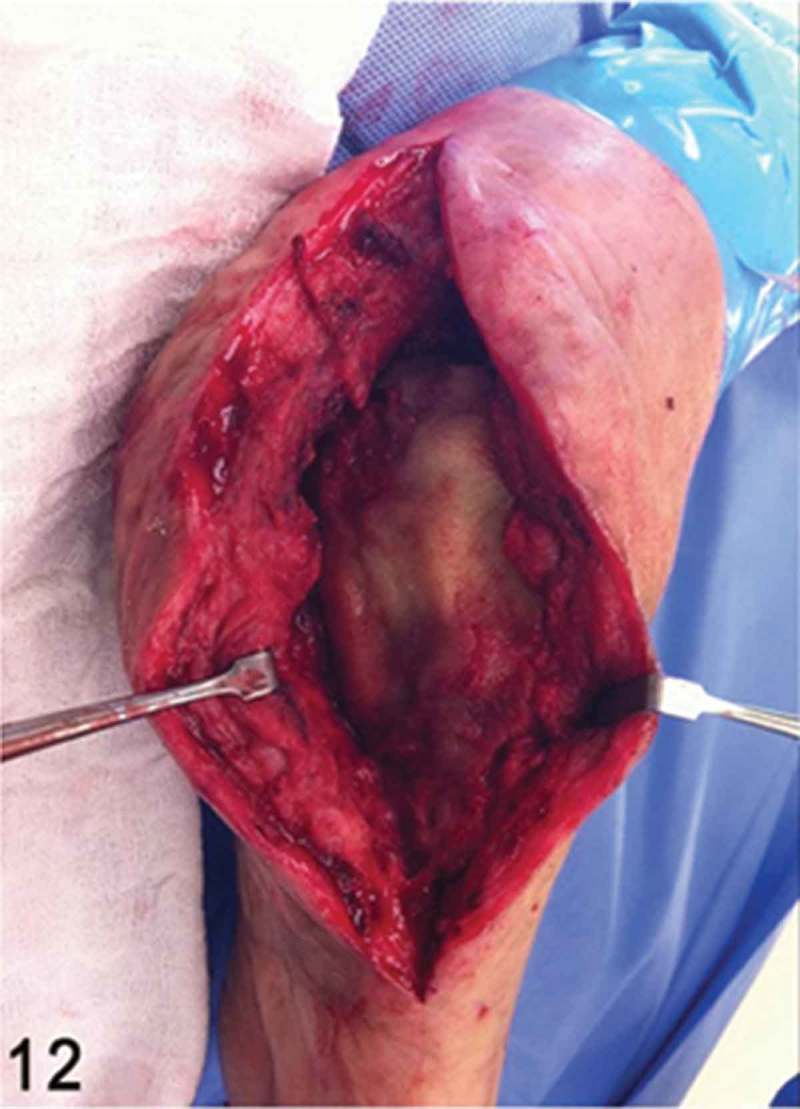
Intraoperative clinical picture after the femoral head bone allograft had been coated with bioactive synthetic bone graft, aiming for a smooth plantar bone surface of the heel.

**Figure 13.**
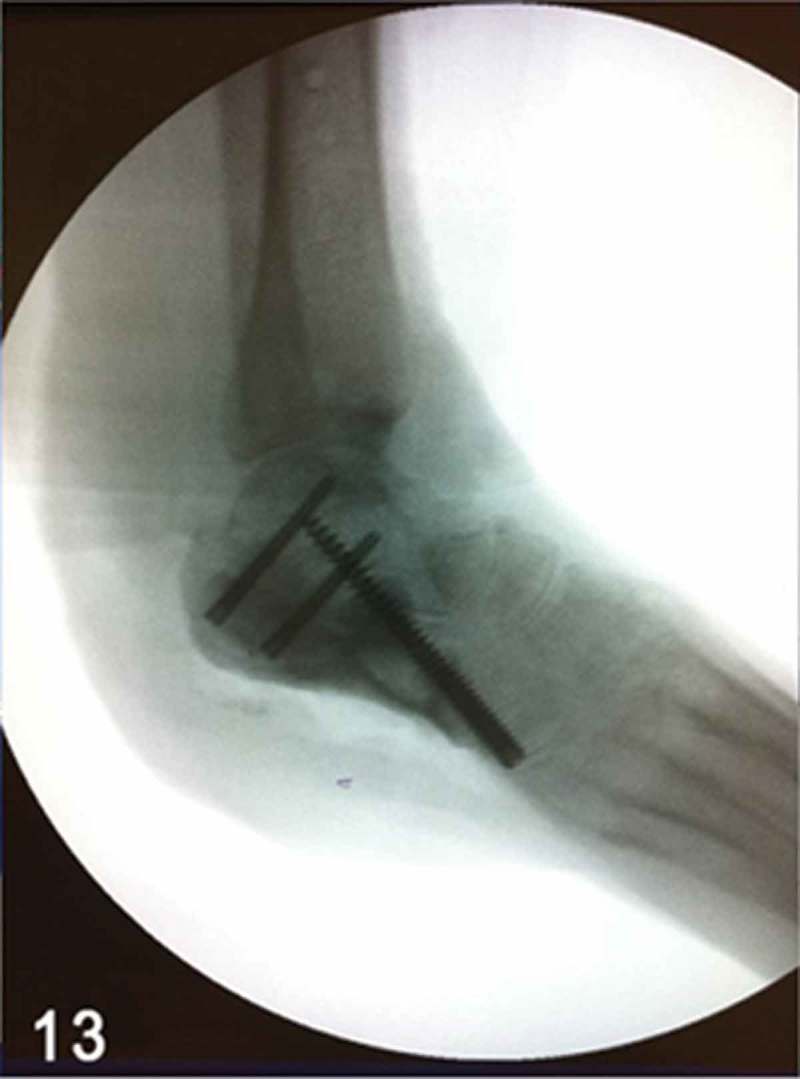
Intraoperative lateral foot fluoroscopic image, demonstrating a more contoured and homogeneous plantar surface of the heel after the bioactive synthetic bone graft had been applied.

**Week 12**: The free flap and split-thickness skin graft had been fully integrated (Figure 8) and the external fixator and bone cement were then removed. We performed a reconstruction of the hindfoot with a femoral head bone allograft coated with bioactive synthetic bone graft (NovaBone®, Bangalore, India). The internal fixation was performed using three Acutrak headless compression screws (Acumed®, Hillsboro, OR, USA) (Figures 9–13).

**Week 20**: The patient initiated a progressive weight-bearing rehabilitation protocol in a long walking boot.

**Figure 14.**
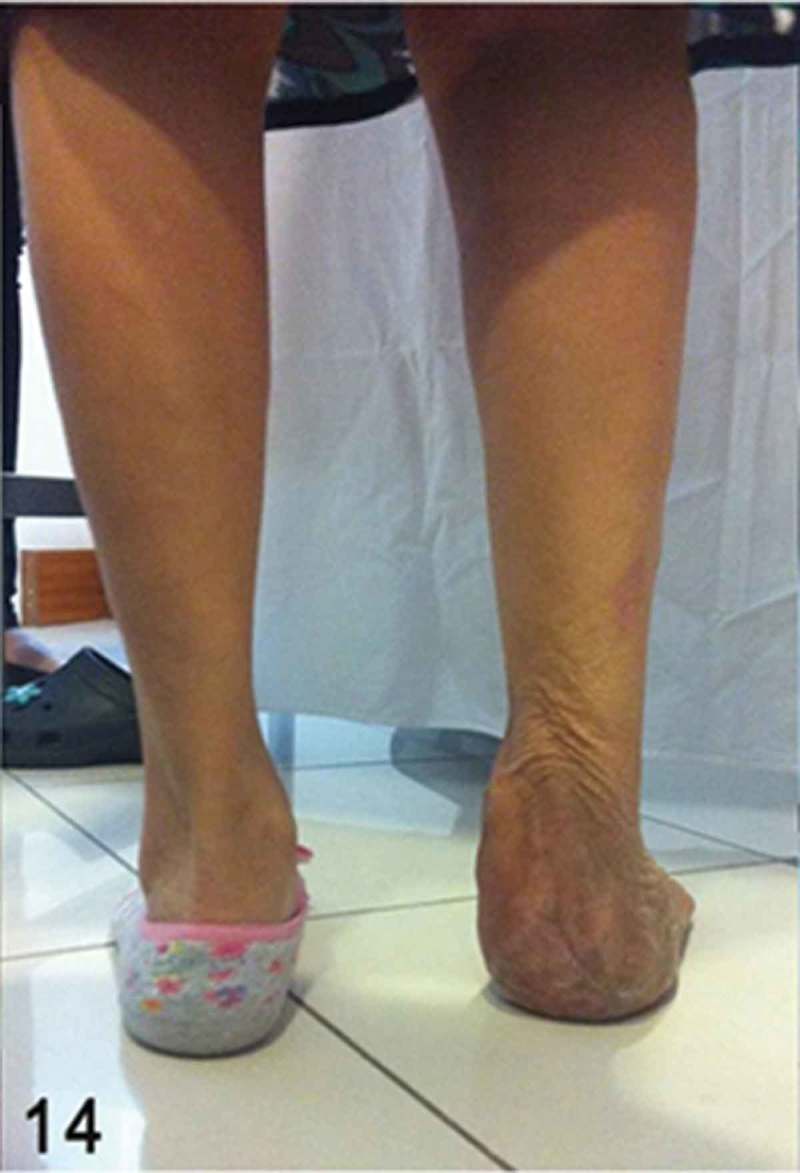
Final clinical outcome. Note the symmetrical alignment in slight valgus of the hindfoot with complete healing of the soft tissue.

**Figure 15.**
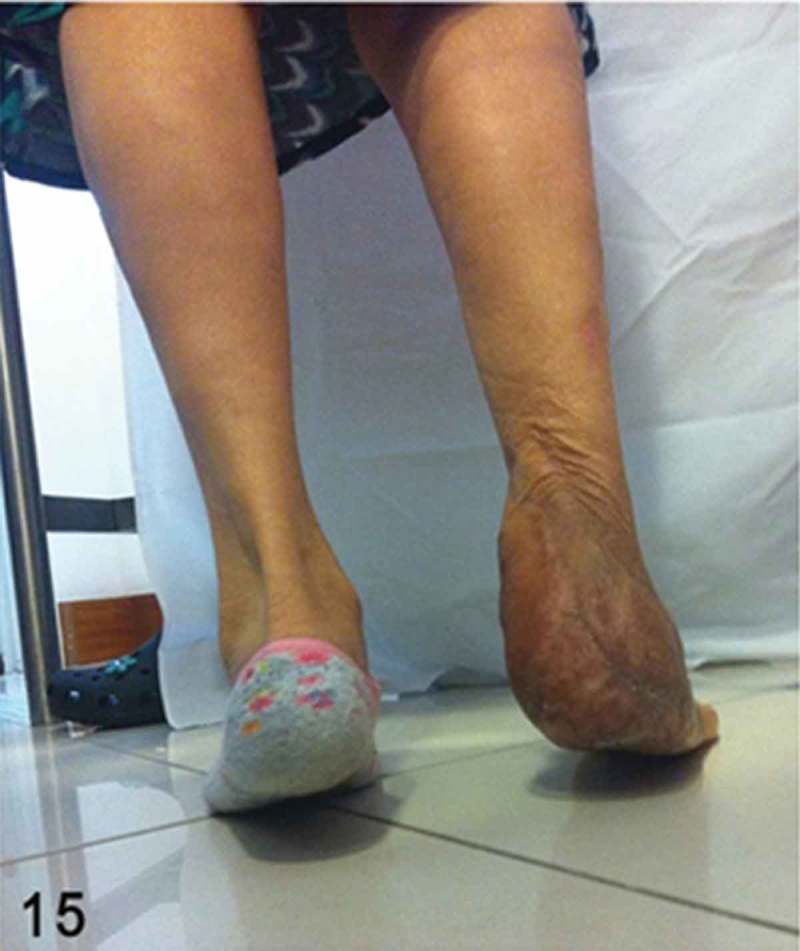
Clinical picture demonstrating relatively preserved plantarflexion strength and competent gastrosoleus muscles when performing a heel raise test.

**Month 36**: The patient was able to walk independently in stiff-soled shoe-gear with an insole compensation for a 0.7 cm shortening in the affected lower extremity. Upon physical examination, a functional full range of motion of the ankle joint, with a 4+/5+ muscle power for dorsiflexion and plantarflexion, was noted (Figures 14 and 15). The patient had also reached 84 points when assessed by the American Orthopaedic Foot and Ankle Society (AOFAS) ankle–hindfoot outcome score.

The authors informed the patient that the data concerning this case would be submitted for publication, and she consented to this.

## Discussion

The initial surgical goals for this patient were similar to damage-control trauma surgery, namely reducing the risk of immediate morbidity and mortality by eliminating the infected and necrotic tissue. Secondary surgical goals after medical optimization include establishing mobility, function, and medium- to long-term quality of life [9,14,16]. For some patients, lower extremity amputation may be a viable option in certain clinical case scenarios. However, this option is often less desirable because of its obvious ablative nature, its psychosocial impact on the patient and family, and the need for aggressive rehabilitation. This leaves a large majority of people for whom diabetic limb salvage may be optimal. Recently, with improvements in vascular, soft-tissue, and musculoskeletal reconstructive techniques, diabetic limb salvage has become a much more viable option.

While negative-pressure wound therapy has almost completely replaced the use of vascularized free flaps as a method for limb preservation, there remains an important role for free tissue transfer [4]. Furthermore, partial calcanectomy can be performed safely and effectively in patients [17], but for those with a high degree of activity, other options such as the one presented here, utilizing bone grafting, may allow for more viable pressure redistribution. In addition, split-thickness skin grafting on the plantar aspect of the high-risk foot, if planned and accommodated with good-quality offloading, can be similarly clinically gratifying [18–20].

## Conclusion

The decision to proceed with aggressive staged efforts at diabetic limb salvage should be taken only after careful consultation with the patient, his or her family, and the rest of the multidisciplinary healthcare team. With the appropriate patient, surgical selection, and communication, the outcomes can often be rewarding long-term results for the patient, family, and clinicians.

## References

[CIT0001] Gregg EW, Li Y, Wang J (2014). Changes in diabetes-related complications in the United States, 1990-2010. N Engl J Med.

[CIT0002] Barshes NR, Sigireddi M, Wrobel JS (2013). The system of care for the diabetic foot: objectives, outcomes, and opportunities. Diabet Foot Ankle.

[CIT0003] Armstrong DG, Wrobel J, Robbins JM. (2007). Guest Editorial: are diabetes-related wounds and amputations worse than cancer?. Int Wound J.

[CIT0004] Franklin H, Rajan M, Tseng CL (2014). Cost of lower-limb amputation in U.S. veterans with diabetes using health services data in fiscal years 2004 and 2010. J Rehabil Res Dev.

[CIT0005] Margolis DJ, Malay S, Hoffstad OJ Incidence of diabetic foot ulcer and lower extremity amputation among Medicare beneficiaries, 2006 to 2008: data points #2. Data Points Publication Series. http://www.ncbi.nlm.nih.gov/books/NBK65149/.

[CIT0006] Kerr M, Rayman G, Jeffcoate WJ (2014). Cost of diabetic foot disease to the National Health Service in England. Diabet Med.

[CIT0007] Ecker ML, Jacobs BS (1970). Lower extremity amputation in diabetic patients. Diabetes.

[CIT0008] Armstrong DG, Lavery LA, Harkless LB (1997). Amputation and re-amputation of the diabetic foot. J Am Podiatr Med Assoc.

[CIT0009] Lavery LA, Van Houtum WH, Armstrong DG (1997). Institutionalization following diabetes-related lower extremity amputation. Am J Med.

[CIT0010] Goldner MG (1960). The fate of the second leg in the diabetic amputee. Diabetes.

[CIT0011] Kucan JO, Robson MC (1986). Diabetic foot infections: fate of the contralateral foot. Plast Reconstr Surg.

[CIT0012] Akhtar S, Ahmad I, Khan AH (2015). Modalities of soft-tissue coverage in diabetic foot ulcers. Adv Skin Wound Care.

[CIT0013] Kallio M, Vikatmaa P, Kantonen I (2015). Strategies for free flap transfer and revascularisation with long-term outcome in the treatment of large diabetic foot lesions. Eur J Vasc Endovasc Surg.

[CIT0014] Fiorito J, Trinidad-Hernadez M, Leykum B (2012). A tale of two soles: sociomechanical and biomechanical considerations in diabetic limb salvage and amputation decision-making in the worst of times. Diabet Foot Ankle.

[CIT0015] Armstrong DG, Lipsky BA (2004). Advances in the treatment of diabetic foot infections. Diabetes Technol Ther.

[CIT0016] Leon LR, Pacanowski J, Ranellone E (2010). Diabetic limb salvage: too much of a good thing?. Vasc Endovascular Surg.

[CIT0017] Fisher TK, Armstrong DG (2010). Partial calcanectomy in high-risk patients with diabetes: use and utility of a ‘hurricane’ incisional approach. Eplasty.

[CIT0018] Rose JF, Giovinco N, Mills JL (2014). Split-thickness skin grafting the high-risk diabetic foot. J Vasc Surg.

[CIT0019] Fitzgerald O’Connor EJ, Vesely M, Holt PJ (2011). A systematic review of free tissue transfer in the management of non-traumatic lower extremity wounds in patients with diabetes. Eur J Vasc Endovasc Surg.

[CIT0020] Bosse MJ, MacKenzie EJ, Kellam JF (2002). An analysis of outcomes of reconstruction or amputation after leg-threatening injuries. N Engl J Med.

